# Occupational Neuroplasticity in the Human Brain: A Critical Review and Meta-Analysis of Neuroimaging Studies

**DOI:** 10.3389/fnhum.2020.00215

**Published:** 2020-07-06

**Authors:** Huijun Wu, Hongjie Yan, Yang Yang, Min Xu, Yuhu Shi, Weiming Zeng, Jiewei Li, Jian Zhang, Chunqi Chang, Nizhuan Wang

**Affiliations:** ^1^School of Biomedical Engineering, Health Science Center, Shenzhen University, Shenzhen, China; ^2^Department of Neurology, Affiliated Lianyungang Hospital of Xuzhou Medical University, Lianyungang, China; ^3^Center for Brain Science and Learning Difficulties, Institute of Psychology, Chinese Academy of Sciences, Beijing, China; ^4^Center for Brain Disorders and Cognitive Science, Shenzhen University, Shenzhen, China; ^5^Lab of Digital Image and Intelligent Computation, Shanghai Maritime University, Shanghai, China; ^6^Department of Electrical and Electronic Engineering, The University of Hong Kong, Pokfulam, Hong Kong; ^7^School of Pharmacy, Health Science Center, Shenzhen University, Shenzhen, China; ^8^Pengcheng Laboratory, Shenzhen, China; ^9^Artificial Intelligence & Neuro-Informatics Engineering (ARINE) Laboratory, School of Computer Engineering, Jiangsu Ocean University, Lianyungang, China

**Keywords:** occupational science, neuroplasticity, occupational neuroplasticity, neuroimaging, meta-analysis

## Abstract

Many studies have revealed the structural or functional brain changes induced by occupational factors. However, it remains largely unknown how occupation-related connectivity shapes the brain. In this paper, we denote occupational neuroplasticity as the neuroplasticity that takes place to satisfy the occupational requirements by extensively professional training and to accommodate the long-term, professional work of daily life, and a critical review of occupational neuroplasticity related to the changes in brain structure and functional networks has been primarily presented. Furthermore, meta-analysis revealed a neurophysiological mechanism of occupational neuroplasticity caused by professional experience. This meta-analysis of functional neuroimaging studies showed that experts displayed stronger activation in the left precentral gyrus [Brodmann area (BA)6], left middle frontal gyrus (BA6), and right inferior frontal gyrus (BA9) than novices, while meta-analysis of structural studies suggested that experts had a greater gray matter volume in the bilateral superior temporal gyrus (BA22) and right putamen than novices. Together, these findings not only expand the current understanding of the common neurophysiological basis of occupational neuroplasticity across different occupations and highlight some possible targets for neural modulation of occupational neuroplasticity but also provide a new perspective for occupational science research.

## Introduction

One of the characteristics of the human brain is its lifelong reorganization of both structure and function (Pascual-Leone et al., 2005). The term “neuroplasticity” refers to changes in function or structure that occur in the brain to adapt to external and/or internal factors (Zilles, 1992; Kempermann, 2006). The extent of neuroplastic remodeling depends on the relevance of individual changes and may have beneficial or maladaptive behavioral consequences (Pascual-Leone et al., 2005). It has been shown that enriched environments and physical activities can increase the growth rate of new neurons and their maintenance in adults (Kempermann et al., 1997; Gage, 2002). However, it is still not quite understood how human brain plasticity happens throughout the life cycle (Johansen-Berg and Duzel, 2016; Walhovd et al., 2016). For example, aging is related to a gradual loss of function in multiple systems, such as the systems underlying sensation, cognition (Kramer et al., 2004), memory (de Lange et al., 2017), motor control, and emotion (Mahncke et al., 2006). In animal models, research indicates that age limits the ability to adapt to changes (Wagner et al., 2000). In contrast, studies have also suggested that older people can quickly learn new skills through training and use them completely unsupervised most of the time (Mahncke et al., 2006; Boyke et al., 2008). The accumulation of age-related brain plasticity will inevitably lead to a growing mismatch between the functional capabilities of older people and what their environment requires of them, as structural plasticity is thought to occur only when demand exceeds capacity (Lövdén et al., 2010) or when there is a considerable change in environment (de Lange et al., 2017). Moreover, an interesting longitudinal study by Brouwer et al. (2017) estimated the heritability of subcortical and global brain volume changes in five sets of twins from geologically different locations and at different stages of life, revealing genetic variants specific to brain plasticity.

Neuroplasticity responds dynamically to environmental changes, and although it is usually beneficial and adaptive, some brain plasticity can be maladaptive in some situations. This paper focuses on the environmental factor of occupation, which subtly and continuously influences the brain through various other long-term and complex factors (e.g., repetition, skilled training, and social relationship) (Falk and Bassett, 2017). External factors act as modulators or inducers of human behaviors that are based on intrinsic structures and activities of the brain. Moreover, according to research findings on neuroplasticity, occupational neuroplasticity has multiple elements, including education, lifestyle, socioeconomic status, social relationships, sustained professional training, and experience. The role of occupational neuroplasticity in the human brain over the life span is not well-understood. Thus, in the following sections, we will fully explore and conclusively infer the neurological mechanisms related to occupational neuroplasticity through a meta-analysis of many published studies. Additionally, the rationality of the neurological findings, the relationship between occupational neuroplasticity and occupational science, and the limitations and future research will be discussed.

## Occupational Neuroplasticity

Professional work styles, activities, abilities, skills, and credentials required by occupations may affect life span development of the brain and the according occupational patterns possibly associated with brain health (Habeck et al., 2019). Studies of occupational neuroplasticity at multiple levels have demonstrated that neural changes can be determined by the significance and structure of the eliciting stimulus On the one hand, neural and behavioral changes attributed to occupational neuroplasticity can happen in several months and extend to the whole lifetime. On the other hand, the stimuli's cortical representations are shaped by occupational experiences continuously on both size and temporal organization. Thus, two related questions come to the forefront: how does the brain shape occupational brain networks, and how does occupation-related connectivity shape the brain? It is difficult to study such effects, but some expert professionals, e.g., athletes, taxi drivers, musicians, dancers, simultaneous interpreters, acupuncturists, seafarers, mathematicians, pilots, and creative writers, represent ideal models in which we can investigate the potential neuroplastic changes in the brain driven by occupations. We will first review the brain changes that characterize the professionals in certain careers, followed by a quantitative meta-analysis of occupational neuroplasticity.

### Occupational Neuroplasticity in Athletes

The brain of athletes provides a suitable model to study neuroplasticity, as they practice throughout their careers, usually beginning early in childhood. A recent study using voxel-based morphometry (VBM) (Ashburner and Friston, 2000) identified clusters in the right cerebellum that had higher gray matter (GM) concentration (GMC) values in experienced badminton players than in novices (Di et al., 2012). Also, this study found a greater cerebellar amplitude of low-frequency fluctuation (ALFF) in the athlete than in the control group, while the ALFF of a cluster in the left superior parietal lobe [Brodmann area (BA)7/BA19] was greater in the control group than in the athletes (Di et al., 2012). Furthermore, a study showed that GM density in the precentral gyrus and left thalamus significantly increased compared with control subjects (Wei et al., 2009). In a comparative study on brain anatomy, cortical thickness of the right parahippocampal gyrus, the right orbitofrontal cortex, and the left superior temporal sulcus was significantly increased in drivers as compared to controls (Sowell et al., 2004; Shaw et al., 2007; Jiang et al., 2009; Wei et al., 2011). Furthermore, there was a significant positive correlation between the number of years of training or the number of years of driving experience and the mean cortical thickness of the right parahippocampal gyrus, which may indicate the impact of long-term professional experience on the structure of the divers' brains (Wei et al., 2011).

Moreover, a study by Kim et al. (2011) has shown that expert archers, as compared with non-archer subjects, showed increased activation in the parahippocampal gyrus, retrosplenial cortex, and cingulate cortex, which are key brain areas of episodic memory and theory of mind-related neural networks. Through fractional anisotropy (FA) analysis of fiber tracts and VBM analyses of white matter (WM) and GM volumes, Jäncke et al. (2009) demonstrated that as compared to non-golfers or less skilled golfers with a handicap from 15 to 36, golfers with a handicap from 1 to 14 and professional golfers have larger GM volumes in the frontoparietal network.

In addition, a variety of functional reorganizations on decision making and body-related processing of the brains of expert basketball players were demonstrated by Abreu et al. (2012), including increased activities in the extrastriate body area during prediction, due to expert observation of action kinematics, and in the right anterior insular cortex and the bilateral inferior frontal gyrus (IFG) when making errors due to awareness of their own errors, while increased activity in the posterior insular cortex during correct action prediction may suggest the importance of body awareness on performance monitoring. Taking world-class athletes (i.e., elite, Olympic, and internationally ranked swimmers) as an example, Huang et al. (2017) found that thalamo-sensorimotor connectivity was significantly correlated with the swimmers' motor performance excellence based on the seed-based functional connectivity (FC) analysis of resting-state functional magnetic resonance imaging data.

### Occupational Neuroplasticity in Taxi Drivers

Extensive navigation experience-related structural and functional changes in the human brain were examined by comparing licensed taxi drivers and controls who were not taxi drivers (Maguire et al., 2003; Woollett et al., 2009; Wang et al., 2015; Shen et al., 2016; Peng et al., 2018). Compared to the controls, taxi drivers showed significantly increased posterior hippocampus volume positively correlated with length of employment, but decreased anterior hippocampus volume negatively correlated with length of employment (Maguire et al., 2000), suggesting local expansion of posterior hippocampus, where the environment was spatially represented, according to navigation experience. Furthermore, in terms of FC changes related to taxi drivers, Wang et al. found that in comparison to the non-drivers, the drivers had reduced intrinsic activity within the visual network and reduced FC between the sensory resting-state networks (RSNs), i.e., the primary and extrastriate visual and sensorimotor ones; additionally, the strength of the FC between the left frontoparietal and primary visual RSNs is positively correlated with length of employment (Wang et al., 2015). Regarding the functional basis of long-term navigation skill, Peng et al. (2018) investigated the role of the entorhinal cortex (EC) and found that taxi drivers had significantly reduced FC between the left anterior–lateral EC and the right anterior cingulate cortex, right angular gyrus, and bilateral precuneus and between the right posterior–medial EC and the left inferior temporal gyrus. Additionally, from the perspective of dynamical connectivity, Shen et al. (2016) found that the vigilance network of taxi drivers showed decreased amplitude of FC fluctuations, with the amplitude being negatively correlated with length of employment.

### Occupational Neuroplasticity in Musicians, Dancers, and Opera Performers

The VBM analysis of Gaser and Schlaug (2003) showed differences in GM volume of the visuospatial, auditory, and motor regions of keyboard player musicians and non-musicians, and similar results were also found by Bermudez et al. (2008) when comparing non-musicians with amateur and professional musicians. They believed that the brains of musicians might have undergone structural changes to adapt to acquisition and rehearsal of professional skills. Furthermore, Bangert et al.study 2006 found increased activity in a distributed cortical network of professional pianists during both silent motion-related and acoustic tasks as compared to non-musicians. This network is composed of Wernicke's and Broca's areas and others.

Compared with non-dancers, female ballet dancers showed decreased GM volumes in the left supplementary, premotor, and motor cortices, as well as the putamen and superior frontal gyrus, decreased WM volumes in both internal capsules, both corticospinal tracts, the corpus callosum, and the left anterior cingulum; and reduced FA in the WM underlying bilateral premotor cortices (Hänggi, 2010). Recently, Lu et al. (2018) found that ballroom dancers, as compared to novices, demonstrated reduced ALFF in the left lingual gyrus; elevated ALFF in the bilateral IFG, bilateral precentral gyrus, left inferior temporal gyrus, left middle temporal gyrus, left middle frontal gyrus, left postcentral gyrus, right superior temporal gyrus (STG), and right middle occipital gyrus; and altered FC among parietal and temporal areas and the IFG.

Neuroplasticity of professional opera performers was studied by Zhang et al. (2018). Professional traditional Chinese Pingju performers, as compared with laymen, demonstrated increased regional homogeneity (ReHo) in the left anterior insula; decreased ReHo in the right middle occipital gyrus, bilateral calcarine, and superior occipital gyri and cuneus; and reduced ALFF in the bilateral cuneus and calcarine gyrus, indicating superior multidimensional performance on dancing, emotional representation, and face and music perception.

### Occupational Neuroplasticity in Simultaneous Interpreters

Simultaneous interpretation makes heavy demands on executive control. In the study of Elmer et al. (2014), it was found that compared to the multilingual controls, the professional simultaneous interpreters had reduced GM volumes in the left supramarginal gyrus, pars opercularis, middle-anterior cingulate gyrus, and bilateral middle insula and pars triangularis, consistent with previous results of negative correlations between length of interpreting experience and GM volume in the bilateral caudate nucleus, left pars triangularis, and right middle-anterior cingulate gyrus and pars opercularis (Ahrens et al., 2010; Elmer et al., 2010). Hervais-Adelman et al. (2014, 2015) compared brain responses observed at both the beginning and the end of a professional training program in simultaneous interpretation, and their results suggested the importance of the caudate nucleus as a central node in networks related to this occupation.

### Occupational Neuroplasticity in Acupuncturists

In a study of brain structural changes among professional acupuncturists, Dong et al. (2013) revealed significantly larger GM volumes in acupuncturists than in non-acupuncturists in the bilateral ventral medial prefrontal cortex/ventral anterior cingulate cortex (VMPFC/vACC), the right cerebellar lobule V/VI, and the left primary somatosensory cortex (SI). Further studies revealed a positive correlation between the duration of acupuncture practice and GM volumes of the left SI and cerebellar V/VI (Dong et al., 2014, 2015). Moreover, in terms of functional alterations, Cheng et al. (2007) were the first to demonstrate that when observing an animation of acupuncture practice, in comparison to naive participants, acupuncturists have increased activation of the temporoparietal junction and the medial and superior prefrontal cortices, which are involved in emotion regulation. Dong et al. (2014) investigated the ReHo alteration of acupuncturists and found that the acupuncturists showed increased ReHo in the left SI, the left primary motor cortex (MI), and the left VMPFC/orbitofrontal cortex. Additionally, Dong et al. (2015) explored the ALFF indicators related to the brain activity of acupuncturists, which demonstrated increased ALFF for acupuncturists in the contralateral hand representation area of the SI and the left VMPFC.

### Occupational Neuroplasticity in Seafarers

Regarding professional seafarers, a resting-state fMRI study by Wang et al. (2017) demonstrated for the first time that seafarers have a distinct atomic connectome pattern (ACP), i.e., ACP14, supporting their vocational requirements; this pattern consists of four specific subnetworks: the visual, auditory, vestibular, and executive control networks. Moreover, Wang et al. (2018) found that the entropy of the STG and orbital–frontal gyrus was significantly higher in seafarers than in non-seafarers, while the cerebellar entropy of the seafarers was lower than that of the controls. The above results imply that seafarers have a more specialized cerebellum and lower capacity for auditory information processing and emotional control than non-seafarers have (Zeng et al., 2016; Wang et al., 2017).

### Occupational Neuroplasticity in Mathematicians

Mathematicians, as members of a highly specialized occupation, require extensive professional training over many years. Aydin et al. (2007) found that mathematicians showed significantly increased cortical GM density in the bilateral inferior parietal and left inferior frontal lobules compared with control subjects. Furthermore, Amalric and Dehaene (2016) found that professional mathematicians showed a reproducibly activated set of bilateral ventrolateral temporal, intraparietal, and frontal regions when facing mathematical statements in geometry, topology, analysis, or algebra. Additionally, Popescu et al. (2019) investigated the structural brain correlates of mathematical expertise, and they found that the mathematicians had increased GM density in the right superior parietal region and decreased GM density in the left IFG and the right intraparietal sulcus. Interestingly, abacus experts (Tanaka et al., 2002) have shown increased brain functional activity in certain cortical areas such as the bilateral superior parietal lobule and superior frontal sulcus that were connected to visuospatial working memory, suggesting that abacus experts utilize visuospatial representations for digital memory.

### Occupational Neuroplasticity in Professional Chess Players

The professional chess player group with long-term training and outstanding logical thinking also provides a suitable model to study the high-level cognition related to cognitive expertise. According to an fMRI study on visual system responding to chess (Krawczyk et al., 2011), it was found that the right temporal cortex, orbitofrontal cortex, and posterior cingulate were more active in chess experts than in novices when observing chess as compared to scrambled chess. Further, the Chinese chess player within resting state showed increased FC between the hippocampus, thalamus, basal ganglia, and parietal/temporal region, denoting the expertise influence on learning- and memory-associated intrinsic connectivity networks (Duan et al., 2014), while in the chess problem-solving task, a broader task-induced deactivation of the default mode network (DMN) in experts was found (Duan et al., 2012). Moreover, a functional network hub analysis was recently performed on the intrinsic FC related to chess experts, which revealed increased FC strength in the right posterior fusiform gyrus as well as its connection to the visuospatial attention and motor networks, in experts over novices (Song et al., 2019). Benefiting from diffusion magnetic resonance imaging (dMRI) studies, structural (WM) differences have been reported in which the bilateral superior longitudinal fasciculus (SLF), inferior longitudinal fasciculus (ILF), and inferior fronto-occipital fasciculus (IFOF) were directly correlated with duration of training in chess experts (Mayeli et al., 2018). Consistently, Feng et al. (2020) detected significant differences in the thalamo-frontal tracts and left SLF. Additionally, a multimodal MRI dataset (29 Chinese chess players and 29 age-matched novices) was released by Li et al. (2015), which could help researchers to further explore underlying neural mechanisms related to chess expertise.

### Neuroplasticity Related to Other Occupations

Ahamed et al. (2014) investigated the brain structural changes of glider pilots in contrast to non-pilots using VBM analysis, which revealed significant increases in GM density in glider pilots in brain areas associated with motor and cognitive processes when piloting a glider, including the left supplementary eye field, anterior cingulate cortex, and ventral premotor cortex. Additionally, Neumann et al. (2018) explored the structural changes in creative writers' brains and found that expert writers had increased GM volumes in the right middle frontal and superior frontal gyri (BA9 and BA10) as well as the left middle frontal gyrus (BA9, BA10, and BA46), left posterior cerebellum, and bilateral medial dorsal nuclei of the thalamus. Interestingly, studies (Kawabata and Zeki, 2004; Vartanian and Goel, 2004) have shown that expertise influences aesthetic judgments, and architects with aesthetic expertise (Kirk et al., 2009) showed differential activation of the bilateral subcallosal cingulate gyrus and medial orbitofrontal cortex compared to non-architects during this judgment. Furthermore, sommeliers (wine experts) (Pazart et al., 2014) also showed activation in the temporal pole and hippocampal and parahippocampal formations when tasting wine; Sreenivasan et al. (2017) have explored the influence of expertise on causal connectivity and topological property related to master sommeliers during different olfactory and non-olfactory tasks, where in sommeliers, a significantly greater connectivity involving the precuneus, caudate, putamen, and several frontal and temporal regions was observed and a significantly higher small-world topology was identified.

In summary, we can see that the occupational factors are indeed able to shape changes in brain structure and function, which are beneficial results of strong occupational neuroplasticity in the human brain.

## Meta-Analysis

Meta-analysis combines a corpus of related studies and extracts concordant findings from them. Activation likelihood estimation (ALE), developed by Turkeltaub et al. (2002), is a most widely used quantitative approach to meta-analysis of neuroimaging studies, utilizing the fact that most functional and structural neuroimaging studies report 3-D (*x, y, z*) coordinates of activity foci in standardized stereotactic space (Laird et al., 2005, 2009; Bzdok et al., 2012; Turkeltaub et al., 2012). For example, Caspers et al. (2010) summarized and amended the previous knowledge of human brain networks, indicating a bilateral network within the parietal, temporal–occipital, and frontal premotor cortices by conjunction meta-analyses of neuroimaging studies on action observation and imitation. Fuelscher et al. (2018) first took advantage of ALE analysis to detect reliable neural correlates of developmental coordination disorder symptoms. Largely based on the recent growth in neuroimaging studies, there are a growing number of suggestions on how best to conduct a meta-analysis (Peyron et al., 2000; Wright et al., 2000; Müller et al., 2018), which have contributed to an integrated view of the brain, and previous studies have provided several methods to analyze enormous functional and structural datasets. In this study, we will we will take advantage of meta-analysis techniques to perform a well-structured meta-analysis of functional and structural neuroimaging studies related to occupational neuroplasticity, respectively.

### Paper Selection and Screening

In this study, we followed the Preferred Reporting Items for Systematic Reviews and Meta-Analysis (PRISMA) guidelines (Moher et al., 2010), and we searched PubMed (http://www.ncbi.nlm.nih.gov/pubmed/) and Google Scholar (https://scholar.google.com) for papers. The keywords included three parts: the research field (e.g., “expert,” “expertise,” “professional,” or “specialist”), the focus on brain functions (e.g., “brain” or “cortical”), and the neuroimaging technique (e.g., “fMRI,” “functional MRI,” or “functional magnetic resonance imaging”). Literature published from 1999 to 2019 was included in this meta-analysis.

In terms of meta-analysis of functional neuroimaging studies related to occupational neuroplasticity, only studies that analyzed local changes in the brain cortex based on fMRI were included in our functional meta-analysis. In total, 26 studies were included, while hundreds of studies had to be excluded for the following reasons:

(1) The studies did not compare experts with novices.
(2) The studies did not report on brain activation as an experimental result.
(3) The studies contained fewer than three stereotactic coordinates or did not report coordinates at all.

**Figure 1:**
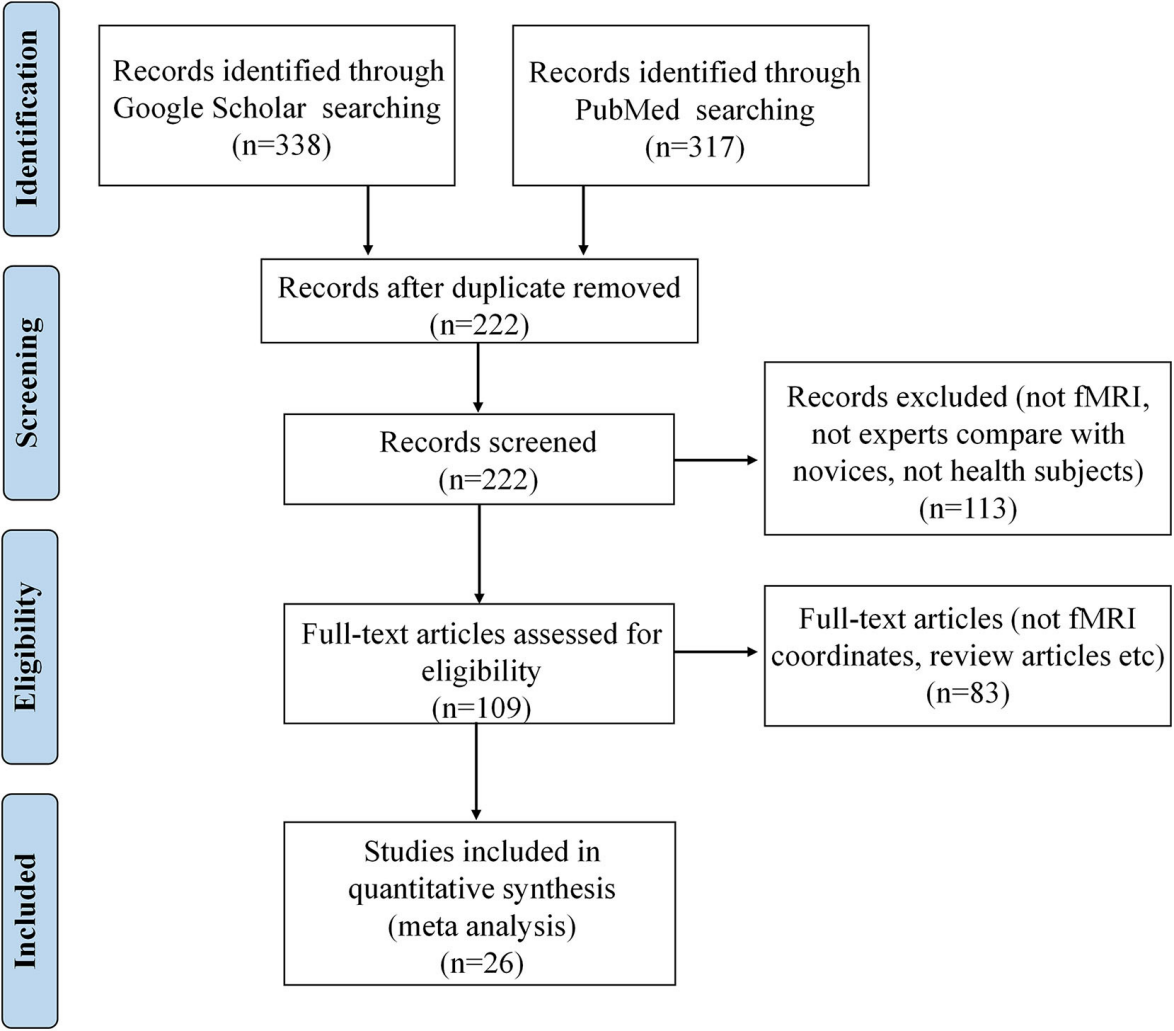
PRISMA flow diagram for eligibility of articles for fMRI meta-analyses on functional activation changes related to occupation.

The corresponding PRISMA flowchart for eligibility of articles for fMRI meta-analyses on functional activation changes was presented in Figure 1, and 26 studies were ultimately included (for details, see Table 1).

**Table 1:** Original fMRI studies included in the functional meta-analysis.

**References**	**Expertise**	**Expert (*N*)**	**Novice (*N*)**	**Task or Rest**
Abreu et al. (2012)	Basketball players	16	16	Motor prediction
Balser et al. (2014)	Tennis players	16	16	Motor prediction
Bangert et al. (2006)	Pianists	7	7	Auditory and motor
Baumann et al. (2007)	Pianists	7	7	Motor execution
Berkowitz and Ansari (2010)	Pianists	13	15	Motor execution
Bishop et al. (2013)	Soccer players	14	11	Motor prediction
Chang et al. (2011)	Archery	18	18	Motor imagery
Di et al. (2012)	Badminton players	20	18	Rest
Duan et al. (2012)	Chinese chess players	15	15	Rest/problem-solving task
Groussard et al. (2010)	Musicians	20	20	Musical semantic memory
Harris and de Jong (2014)	Keyboard players	12	12	Motor imagery
Haslinger et al. (2004)	Pianists	12	12	Motor execution
Kim et al. (2011)	Archery	20	21	Motor observation
Koeneke et al. (2004)	Keyboard players	7	7	Motor execution
Krawczyk et al. (2011)	Chess players	6	6	Face processing
Lee and Noppeney (2011)	Pianists	18	19	Music listening
Lotze et al. (2003)	Violinists	8	8	Motor execution and imagination
Luo et al. (2012)	Musicians	15	15	Motor and multi-sensory
Pilgramm et al. (2010)	Dancers	18	18	Motor observation
Seo et al. (2012)	Archery	20	23	Visuospatial memory
Song et al. (2019)	Chess players	28	27	Rest
Tomasino et al. (2013)	Volleyball players	10	10	Motor language processing
Wang et al. (2018)	Seafarers	20	20	Rest
Wright et al. (2011)	Badminton players	8	8	Motor prediction
Wright et al. (2013)	Soccer players	17	17	Motor observation
Xu et al. (2016)	Badminton players	16	18	Motor prediction

In terms of meta-analysis of structural neuroimaging studies related to occupational neuroplasticity, we also conducted a comprehensive search for structural MRI morphometric studies on PubMed and Google Scholar (search strings: “player + morphometry,” “player + brain + mri,” “player + brain + VBM,” “expert + morphometry,” “expert + brain + mri,” and “expert + brain + VBM”). Literature published from 1999 to 2018 was included.

Only studies that analyzed local changes in GM based on structural MRI were included in our structural meta-analysis. In total, 17 studies were included, and some studies were excluded due to the following reasons:

(1) The studies did not compare experts with novices.
(2) The studies reported only volumetric data.
(3) The studies contained fewer than three stereotactic coordinates or did not report coordinates at all.

**Figure 2:**
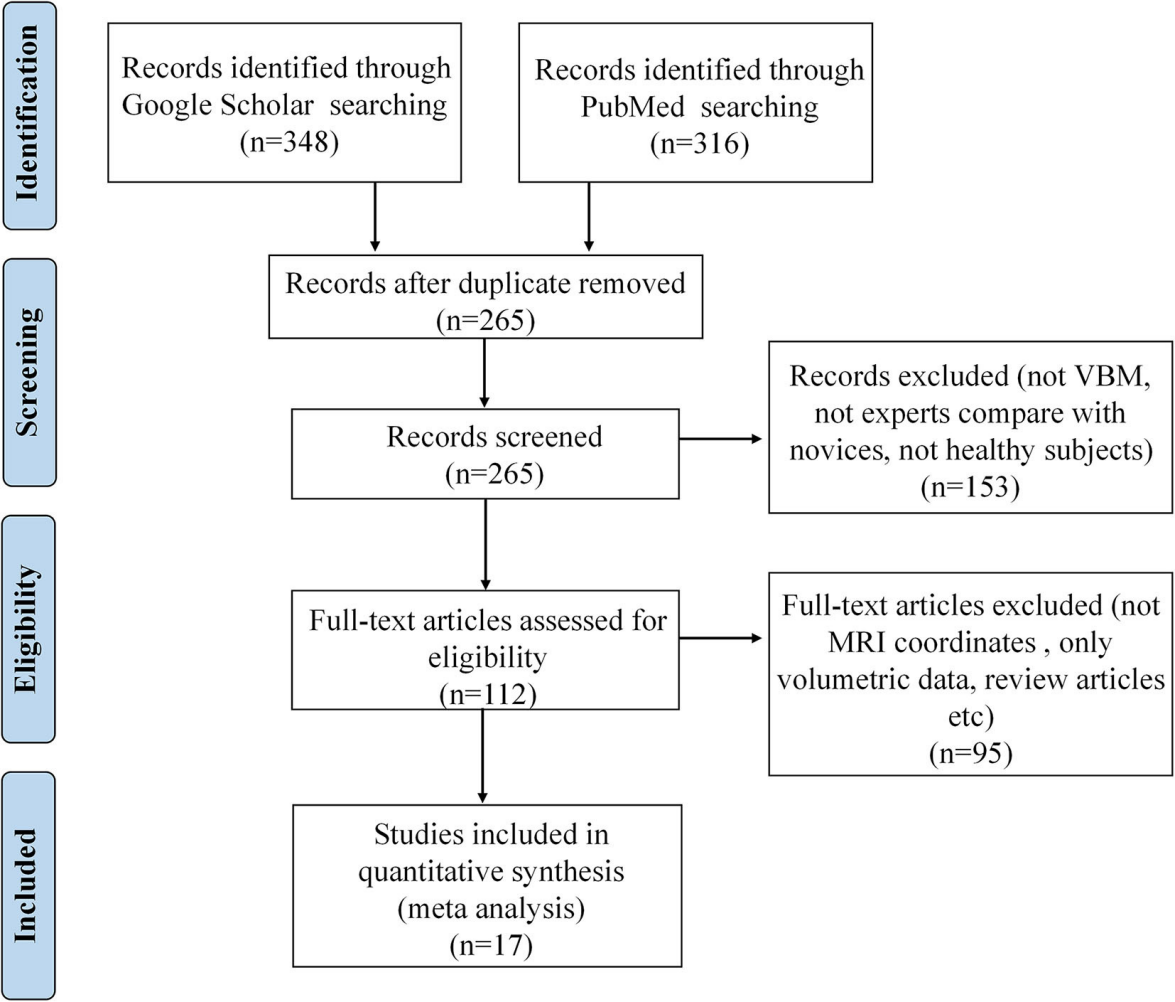
PRISMA flow diagram for eligibility of articles for MRI meta-analyses on structural volume changes related to occupation.

The corresponding PRISMA flowchart for eligibility of articles for MRI meta-analyses on volume changes was presented in Figure 2, and 17 studies were finally included (for details, see Table 2).

**Table 2:** Original MRI studies included in the structural meta-analysis.

**References**	**Expertise**	**Expert (*N*)**	**Novice (*N*)**
Ahamed et al. (2014)	Glider Pilots	15	15
Bermudez et al. (2008)	Musicians	71	64
Di et al. (2012)	Badminton Players	20	18
Fauvel et al. (2014)	Musicians	16	17
Groussard et al. (2014)	Musicians	22	22
Hänggi (2010)	Ballet Dancers	10	10
Hernández et al. (2016)	Sahaja Yoga Meditators	23	23
Hu et al. (2018)	Track Athletes/Badminton Players	13/13	16
Huang et al. (2015)	Gymnasts	13	13
Hüfner et al. (2011)	Dancers & Slackliners	21	20
Jäncke et al. (2009)	Golfers	20	20
James et al. (2014)	Musicians	18	19
Kleber et al. (2016)	Opera Singers	27	28
Li et al. (2011)	Musicians	15	15
Li et al. (2018)	Musicians/Dancers	20/18	25
Tan et al. (2017)	Basketball Players	21	21
Vaquero et al. (2016)	Pianists	36	17

### Analytical Procedure

GingerALE 2.3.6 (UT Health Science Center Research Imaging Institute, San Antonio, TX) was used as an implementation of ALE quantitative meta-analysis of neuroimaging studies with a random-effects model, combined with cluster-level inference with a false discovery rate (FDR) (Genovese et al., 2002) of *q* = 0.05.

Coordinates of peak voxels in those studies originally reported in the Talairach system were converted to the Montreal Neurological Institute (MNI) system using the WFU PickAtlas software (Maldjian et al., 2003). Multi-Image Analysis GUI (“Mango,” UT Health Science Center Research Imaging Institute) was used to display our ALE meta-analytic findings (see Results section below).

## Results

### Brain Activation Changes Related to Occupation

#### Functional Contrast: Experts > Novices

**Figure 3:**
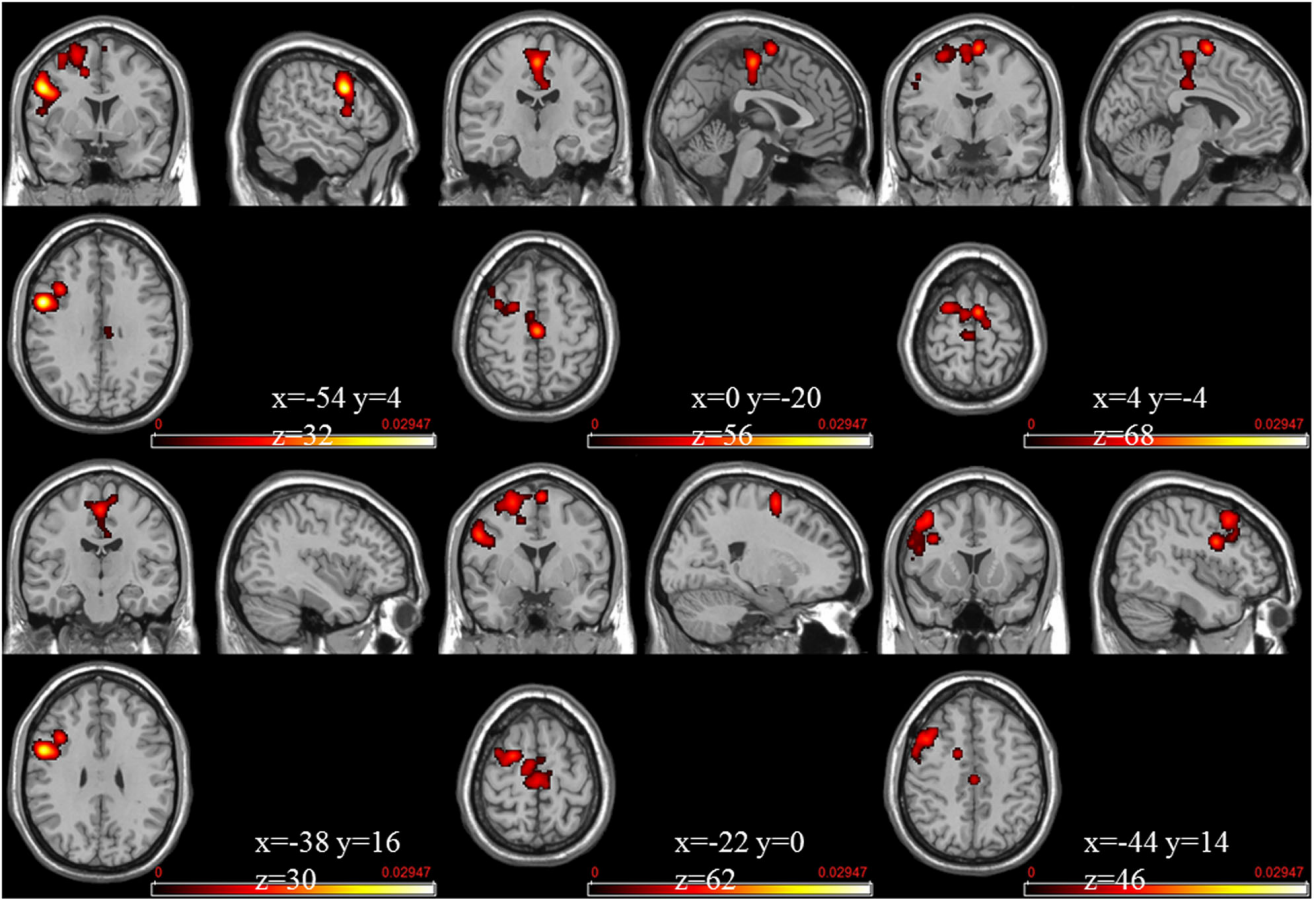
Strengthened activation estimated by ALE with cluster-level correction (FDR of *q* = 0.05) in experts compared with novices.

**Figure 4:**
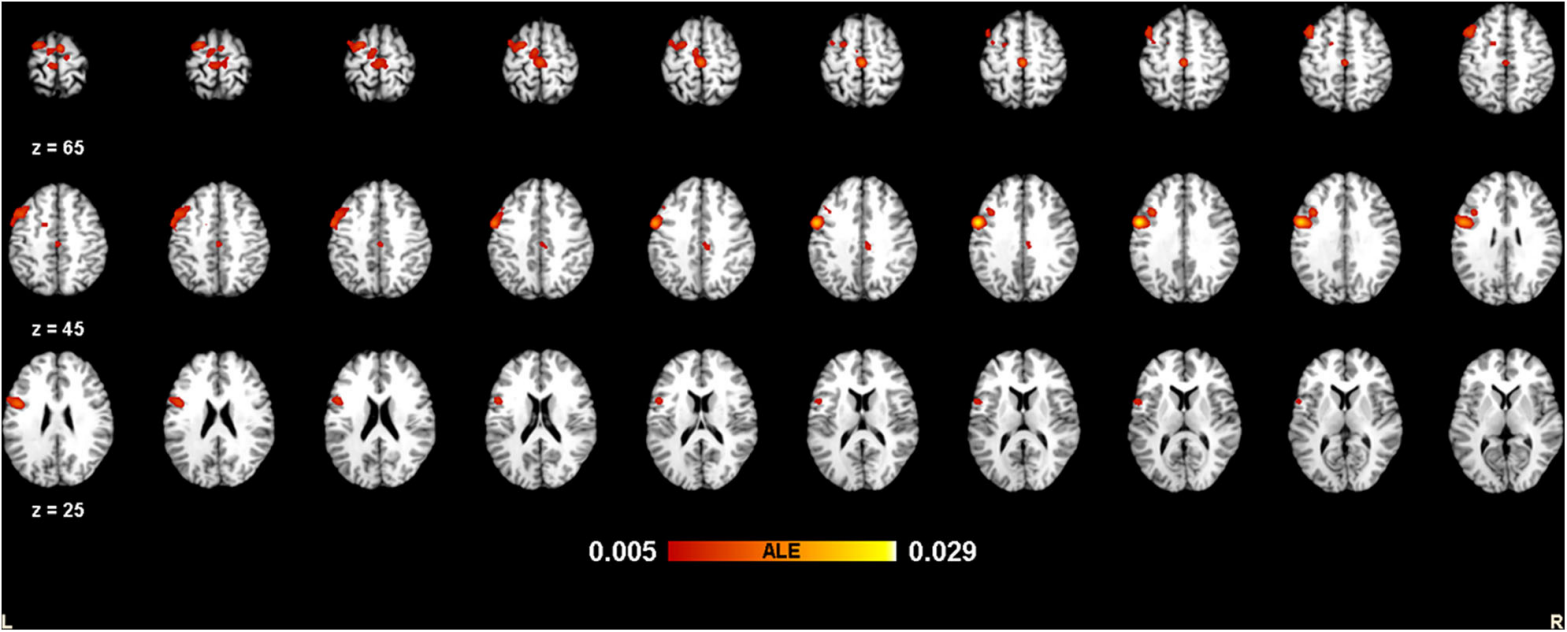
Strengthened activation estimated by ALE with cluster-level correction (FDR of *q* = 0.05) in experts compared with novices, shown in a multislice view.

The meta-analysis of functional activation changes in this contrast included 26 studies with 365 foci. The results indicated six significant clusters located in BA6 and BA9, which are shown in Table 3 and Figures 3, 4.

**Table 3:** Strengthened activation results in experts compared with novices in functional neuroimaging meta-analysis.

**Volume (mm^**3**^)**	***x***	***y***	***z***	**Label**	**Brodmann area**
707	−54	4	32	L Precentral Gyrus	BA6
160	0	−20	56	L Paracentral Lobule	BA6
641	4	−4	68	R Medial Frontal Gyrus	BA6
210	−38	16	30	L Precentral Gyrus	BA9
833	−22	0	62	L Middle Frontal Gyrus	BA6
572	−44	14	46	L Middle Frontal Gyrus	BA6

### Structural Volume Changes Related to Occupation

#### Structural Contrast: Experts > Novices

**Figure 5:**
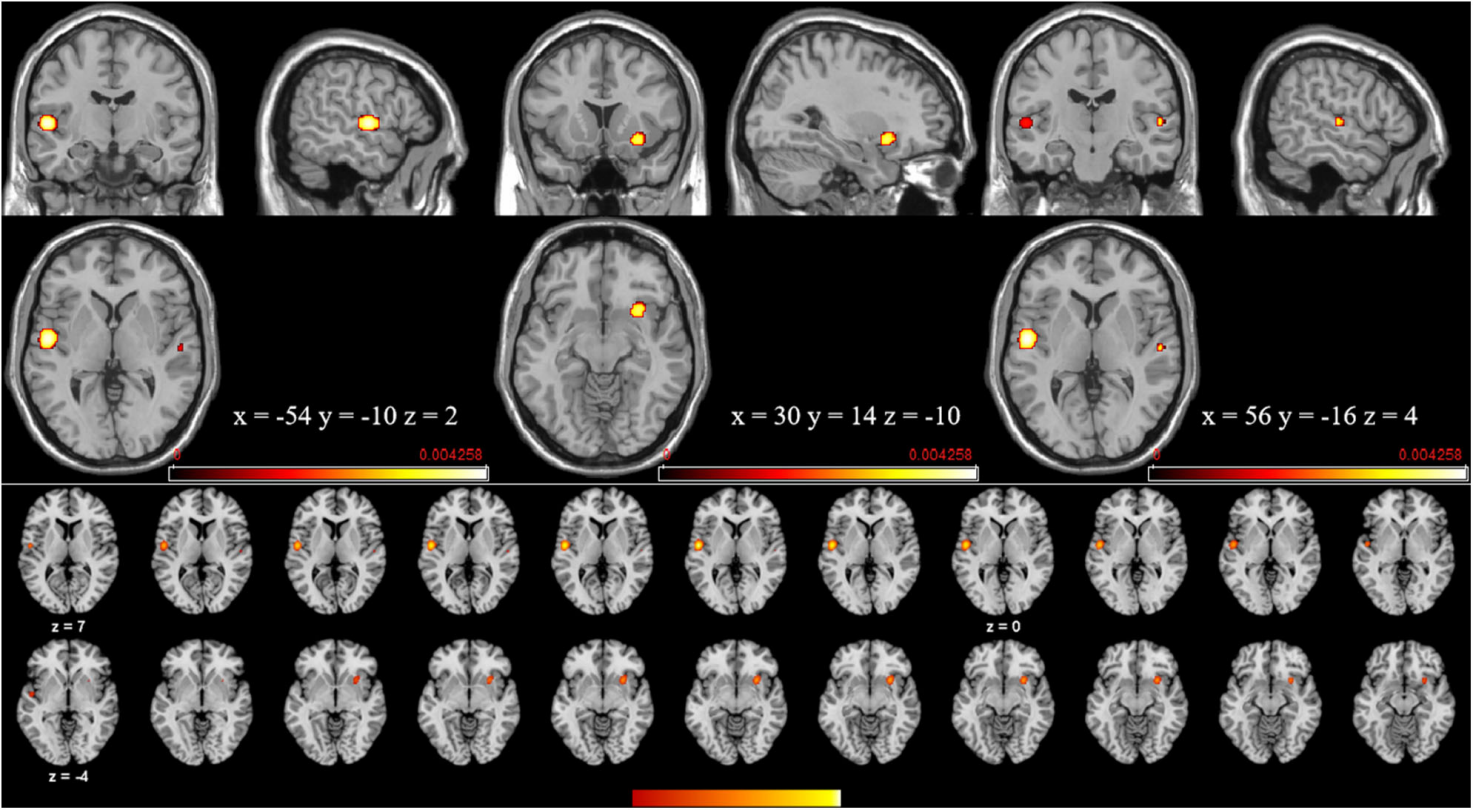
Volume increases estimated by ALE with cluster-level correction (FDR of *q* = 0.05) in experts compared with novices.

The meta-analysis of structural changes in this contrast included 17 studies with 101 foci. The results indicated three significant clusters located in the right putamen, the left STG (BA22), and the right STG (BA22), which are shown in Table 4 and Figure 5.

**Table 4:** Volume increase results in experts compared with novices in structural neuroimaging meta-analysis.

**Volume (mm^**3**^)**	***x***	***y***	***z***	**Label**	**Brodmann area**
1984	−52	−8	2	L Superior Temporal Gyrus	BA22
1200	28	12	−10	R Putamen	–
80	56	−16	4	R Superior Temporal Gyrus	BA22

## Discussion

### Occupational Science and Occupational Neuroplasticity

Occupational science is the study of human occupation, which has two elements, i.e., the human and the occupation (Hocking, 2009). The central concept within occupational science is occupation itself, which is mainly characterized by diversity. A conceptual research framework with respect to occupational science consists of the essential elements of occupation and occupational processes as well as the relationship between occupation and other phenomena (Hocking, 2000). In terms of the essential elements of occupation, the focus of research is to identify the nature, structure, substrates, and features of occupation. With respect to occupational processes, the focus of research is to investigate the process, outcomes, features, and subjective experience of occupational performance. Considering the relationship between occupation and other phenomena, the focus of research is to explain how occupation relates to identity, health, human development, quality of life, and social structures and policies (Hocking, 2000). As seen from the above, occupation and the related occupational science are very complex, diverse, and comprehensive.

The concept of “occupational neuroplasticity,” as introduced in this paper, can be treated as a potential branch of occupational science that focuses on disclosing the relationship between the brain's plasticity (functional neuroplasticity, structural neuroplasticity, etc.) and long-term occupational training or experience. Due to the diversity and complexity of occupation, occupational neuroplasticity shows the heterogeneity among many different types of occupation, with a complex intrinsic mechanism and an ongoing process of comprehensive change related to occupational development and other factors such as health status, mental illness, and neuropsychiatric or neurological disease. Specifically, as mentioned before, occupational neuroplasticity is associated with the process of acquiring professional skills through sustained performance. This process comprises multiple elements, i.e., professional acquisition, interpersonal relationships, social reward and competition systems, consistent work environments, and possible genetic factors; these numerous elements make the process very complex and comprehensive. Taking environmental factors as an example, toxins, drugs, genetic mutations, nutritional disturbances, and environmental sensory and behavioral conditions all affect neuronal network formation at multiple levels in the developing brain (Fulton, 1935). This might provide new perspectives into the mechanisms of occupational neuroplasticity, where various occupational environments cause both benefits and drawbacks and are closely related to mental health, aging, mental disorders, neurological diseases, neurodegenerative diseases, etc. Thus, the exploration of occupational neuroplasticity can not only help uncover the commonly intrinsic neurophysiological mechanisms shaped by occupation (the main focus of this paper) but also accumulate knowledge and data on mental health promotion, prevention, and intervention in occupational therapy (Haglund and Henriksson, 2003; Arbesman et al., 2013; Read et al., 2018).

### Functional Neuroplasticity Driven by Occupation

Through the meta-analysis of functional neuroimaging studies related to occupation, we found that the cortical regions BA6 and BA9 were significantly related to the occupational experience training, providing some functional evidences of occupational neuroplasticity, as we discussed below.

We will begin by discussing BA6, a traditional “motor” area that many studies suggest to be active during higher motor control involving a variety of cognitive operations (Fulton, 1935; Wise, 1985; Freund, 1990). Some neuroanatomical evidence suggests that although the caudal region of BA6 is closely related to the primary motor cortex and produces a large number of corticospinal projections, the rostral region of BA6 is closely related to the prefrontal cortex rather than the primary motor cortex, sending no direct projections to the latter (Barbas and Pandya, 1987; Luppino et al., 1993; Lu et al., 1994). However, research results on this topic are inconsistent, and the structural and functional roles of BA6 in cognition and in motor control are unclear (Picard and Strick, 2001; Schubotz and von Cramon, 2003). Functional activation in BA6 during cognitive tasks is often interpreted as accompanying potential motor processes, such as preparation for button pressing or eye movement; therefore, it is not out of the question for the functional relevance of BA6 activation in cognition (Courtney et al., 1998). Furthermore, BA6 has modality-specific cognitive functions (Tanaka et al., 2005), and there is growing evidence that some classically designated “motor” regions also take functions in non-motor cognitive processes (Ito, 1993; Leiner et al., 1993; Middleton and Strick, 1994; Tanaka et al., 2005). Additionally, convergent pieces of evidence from neuroimaging studies suggest a unique role for part of BA6, i.e., the medial frontal cortex, which is closely related to social cognitive processing (Amodio and Frith, 2006). Considering the complexity and diversity of occupation and the results found in this study, long-term experience with occupational training potentially causes the significant alternations in motor control, non-motor cognitive function, and social cognitive processing among experts in contrast to novices.

The region of BA9 focused on in this paper, i.e., the IFG, is considered to be related to general cognitive function, which is involved in working memory (Zhang et al., 2003; Schulze et al., 2011), cognitive control of memory (Badre and Wagner, 2007), selection or inhibition of other self-control functions (Goghari and MacDonald, 2009), cognitive flexibility (Ghahremani et al., 2009), speech and language processing (Greenlee et al., 2007), inhibitory processes (Sharot et al., 2012), etc. This suggests that long-term occupational training experience is a general, comprehensive, ongoing training process that is associated with many factors in occupational training. Furthermore, the strengthened effects in experts in the right IFG of BA9 and the left precentral gyrus (BA6), part of the mirror neuron system (Rizzolatti et al., 2014), suggest that these areas become more involved in imitating other people's movements to understand the corresponding emotions and intentions after long-term occupational training (Molnar-Szakacs and Overy, 2006) in one's career. Indeed, according to previous studies, experts have a larger left frontal area than controls (Sluming et al., 2002), and GM density increases with musical expertise (Gaser and Schlaug, 2003; James et al., 2014).

According to the aforementioned discussion, due to the complexity, diversity, and comprehensiveness of occupation, the long-term training required by one's occupation may create specifically intrinsic representations to match the demands of one's own career, involving functions such as sensation, movement, non-motor cognition, social cognitive processing, working memory, and speech and language processing.

### Structural Neuroplasticity Driven by Occupation

Many studies have suggested that structural neuroplasticity may evolve in all directions under influence modulation of certain skill learning and expertise (Chang, 2014). In this present study, as compared to novices, experts showed greater GM volume in the right putamen, classically associated with motor control and more recently with memory-related processes, reinforcement learning, and implicit sequence learning (Vaquero et al., 2016). This phenomenon may imply that the functions of movement, learning, and memory are very important in the occupational training process. Another cortical area with a larger GM volume in experts than in novices was the STG (BA22), which contains the primary auditory area (Wernicke's area) and shows a key role in language processing. The left STG region is involved in the understanding and generation of individual words, while the right STG is involved in discriminating sound intensity and pitch. Additionally, the left auditory cortex may process rapid changes in temporal properties more precisely than the right auditory cortex (Schneider et al., 2002; Zatorre, 2013). Thus, according to the previous discussion of structural neuroplasticity, we can speculate that occupation-related training or experience possibly impacts the capacity for auditory and language processing, motor functions, learning, and memory, which play key roles in carrying out the functions of one's occupation and provide some structural evidence of occupational neuroplasticity.

### Target Areas for Occupational Neuroplasticity Modulation

In this research, some common regions (see Figures 3–5) were robustly identified by meta-analysis in terms of occupational neuroplasticity across a lot of occupations, which demonstrates a common neural mechanism of occupational neuroplasticity. Further, these identified common regions in terms of structural or functional changes possibly are treated as the target ones in daily and specifically designed occupational training activities and through neuromodulation approaches, i.e., transcranial magnetic stimulation (TMS) (Pascual-Leone et al., 2002), transcranial direct current stimulation (tDCS) (Paulus, 2011), etc., which possibly improve the occupational capability. For example, Reis et al. (2008) reviewed many related papers and found that the aforementioned stimulation techniques could regulate memory formation and motor learning in healthy humans. Also, many researches demonstrated that the above stimulation techniques could be an effective treatment for psychiatric disorders (e.g., major depressive disorder) (Reis et al., 2008; Liu et al., 2017) and neurodegenerative dementias (Elder and Taylor, 2014). Thus, based on previous studies, the identified portions (listed in Tables 3, 4) of BA6, BA9, and BA22 are likely located as targets in neural modulation of occupational capability at the individual or group level.

### Inconsistency of Structural and Functional Alterations Concerning Occupation

According to the presented meta-analysis results about the brain structure and function regarding occupation, the inconsistent alterations were observed, which may result from the following points. At the beginning, the included papers in structural and functional meta-analyses were not identical, because the results of both functional and structural alterations regarding a certain occupation were less reported simultaneously in a paper. This brought great subject diversity in the structural meta-analysis and functional meta-analysis. Further, the reported VBM results from the included papers quite depended on the resolution of T1 brain images, where magnetic field intensity had obvious main effects (Obusez et al., 2018). The recently developed quantitative MRI (qMRI) with its robustness and effectiveness could be an alternative to investigate the brain structure changes corresponding to occupation in the future (Weiskopf et al., 2013). In addition, this phenomenon likely originates from the intrinsic inconsistency between anatomical connectivity and FC (Park and Friston, 2013; Messé et al., 2014). For example, Park and Friston (2013) pointed out that the divergence of function from structure is the most intriguing property of the brain; Messé et al. (2014) also revealed that anatomical connectivity alone accounts for up to 15% of FC variance. To sum up, exploring the connection between the functional neuroplasticity and structural neuroplasticity with regard to occupation is a key part of occupational neuroplasticity in future studies.

### Limitations and Future Research

The present investigation has some limitations. First, due to the heterogeneity and diversity of occupation, there are few studies on certain types of occupations, which imposed some restrictions on exploring the mechanism at the individual occupation level by means of meta-analysis. Thus, in the future, we advocate more studies on occupational neuroplasticity, which contributes to the understanding of the intrinsic mechanism of occupational neuroplasticity at the group or individual occupation level. Second, in this paper, we reported some neuroimaging results that included only the changes of GM and functional activation due to lack of WM studies; extensive research on occupational neuroplasticity could remedy this lack of data on WM. Third, the relationship between occupational neuroplasticity and the duration of occupational experience also can be further investigated. In addition, how the level of work complexity is closely related to the occupational neuroplasticity should be addressed in the future. Finally, whether occupational neuroplasticity is predominantly determined by nature (internal genes) or nurture (external experimental factors) or both remains an open question and needs to be explored carefully in future studies.

## Conclusion

Advances in neuroimaging over the past few decades have improved our understanding on the neuroplastic changes due to skill learning, expertise, etc., in terms of occupation. Based on the meta-analysis, these plastic changes can be demonstrated clearly at structural and functional levels (Figures 3–**5**), suggesting the possible common neurophysiological basis of occupational neuroplasticity. In terms of functional reorganization, a common finding in experts is a set of changes mainly centered on activation of brain cortical areas BA6 (mainly including the medial frontal gyrus, middle frontal gyrus, precentral gyrus, and paracentral lobule) and BA9 (including the IFG), likely in order to satisfy occupational requirements by strengthening functions such as sensation, movement, non-motor cognition, social cognitive processing, working memory, and speech and language processing. Meanwhile, in terms of structural reorganization, structural analysis reveals that experts show increased GM volume in the cortical region BA22 (included in the STG) and in the putamen, possibly supporting occupationally necessary abilities such auditory and language processing, movement, learning, and memory. The outlined areas could be used as the likely targets for modulating occupational neuroplasticity, aiming at improving the comprehensive occupational capability and further revealed the commonly or basically underlying neural mechanism across different kinds of occupation. Moreover, we discussed the rationality of the occupation-related functional and structural differences discovered in experts compared with novices and the inconsistent alterations of structural and functional reorganization concerning occupation factor. Finally, we thoroughly reviewed pieces of evidence related to occupational neuroplasticity in many types of occupations, such as athletes, taxi drivers, musicians and dancers, simultaneous interpreters, acupuncturists, seafarers, mathematicians, pilots, and creative writers. In summary, our findings enriched the conceptualization of neuroplasticity, partly defined the neural basis of occupational neuroplasticity, and provided a new perspective for occupational science research.

## Data Availability Statement

All datasets generated and analyzed for this study are included in the article/supplementary material.

## Author Contributions

HW and HY analyzed the data and wrote the draft. NW conceived of the study, checked the results, and wrote the paper. YY, MX, YS, WZ, JL, and JZ discussed the study, read the manuscript, and gave feedback. CC discussed the study extensively, read and revised the draft, and provided commentary. All authors contributed to the article and approved the submitted version.

## Conflict of Interest

The authors declare that the research was conducted in the absence of any commercial or financial relationships that could be construed as a potential conflict of interest.
